# Bio-Degradable Polyurethane Foams Produced by Liquefied Polyol from Wheat Straw Biomass

**DOI:** 10.3390/polym12112646

**Published:** 2020-11-10

**Authors:** Luis Serrano, Esther Rincón, Araceli García, Jesús Rodríguez, Rodrigo Briones

**Affiliations:** 1 Inorganic Chemistry and Chemical Engineering Department, University of Cordoba, 14014 Cordoba, Spain; b32rirue@uco.es; 2 Organic Chemistry Department, University of Cordoba, 14014 Cordoba, Spain; qo2ganua@uco.es; 3 Centro de Investigación de Polímeros Avanzados (CIPA), Av. Collao, Concepción 1202, Chile; j.rodriguez@cipachile.cl (J.R.); r.briones@cipachile.cl (R.B.)

**Keywords:** wheat straw, liquefaction, foams, biodegradability

## Abstract

In the present work, an abundant and unused residue (wheat straw) has been employed to synthesize a polyol as a substituent of castor oil in polyurethane foams. The liquefied product showed excellent properties for the proposed application. Castor oil was substituted with up to 50% wheat straw polyol in the formulation of polyurethane foams, which were prepared using two different isocyanates (methylene diphenyl diisocyanate (MDI) and toluene-2,4-diisocyanate (TDI)). The evaluation of physical, mechanical, and thermal properties of the foams revealed that these materials can successfully be formed with up to 40% wheat straw polyols since all the results were improved. Moreover, at this polyol concentration, the morphology of the foams was presented as a compact and ordered structure. Following this trend, the foams showed excellent biodegradability at 30 days (5.60 and 7.31% for TDI and MDI foams, respectively) and 60 days (8.49 and 9.88% for TDI and MDI foams, respectively) in the soil media tests carried out. Thus, the materials prepared in this work can be proposed for agricultural applications such as use in plant nurseries.

## 1. Introduction

The growing demand of the population on renewable energy sources, which overcome the dependence on fossil fuel resources, has led to many efforts to find the optimal source that meets expectations. In this regard, crop residues represent more than half of the agricultural biomass worldwide. Usually, these residues are discarded. Therefore, an efficient use of this biomass could solve the problems mentioned above [1].

Wheat is an agricultural crop with one of the highest production rates (734 million tons) and largest cultivation areas (214 million hectares) worldwide [2]. Therefore, it is also one of the major sources responsible for the generation of lignocellulosic agricultural biomass [3]. Specifically, if it is considered that 1 kg of grain produces 1 kg of straw (residue), an enormous amount of waste is generated each year [4]. Many efforts have been made in recent years to valorize this residue and these studies comprise a variety of applications such as bioethanol production [5], biocomposite obtention, food packaging materials acting as a polymer matrix [6,7], application as a binder in lithium batteries [8], and as part of polymer blends [9]. These successful examples of applications for wheat straw are very encouraging for the continuation of research towards new ways of exploitation. 

In addition to these lignocellulosic biomass recovery strategies, there is another option known as liquefaction. That is, to carry out the complete conversion of the biomass into liquid fuels without the gasification step [10]. Thermochemical conversion mechanisms such as liquefaction allow the conversion of plant biomass into interesting products such as heat, power, electricity, chemicals, fuels, and other high-value materials. Traditionally, not much attention was paid to liquefaction as the requirements were complex and expensive, making it difficult to scale up industrial processes. However, advances in this field have made it possible to increase reaction yields by using organic solvents at atmospheric pressure and low temperature, thus obtaining the desired polymers and chemicals [11]. In particular, the liquefaction products that have been of most interest are bio-based adhesives and polyols. This is because they are compatible with conventional adhesives and resins such as epoxides, polyurethanes, and isocyanates, materials used in industries such as construction, automotive, pharmaceutical, etc., and which usually come from petroleum derivatives [11]. Of these, polyols are of particular importance since they are capable of being combined with isocyanates for the production of polyurethanes, used to produce flexible polyurethane foams, rigid polyurethane foams, coatings, adhesives, sealants, and elastomers [12]. 

Polyurethane foams are usually prepared by reacting an isocyanate compound and a polyhydroxyl-containing polymer. The latter is commonly derived from petrochemicals [13]. However, in order to partially or totally substitute these oil-based polyols, a wide variety of vegetable oils are being proposed, such as sunflower, castor, and soybean oils [14]. Castor oil is a very versatile vegetable oil due to its chemical composition, whose main component is 12-hydroxy-9-*cis*-octadenoic acid. This makes castor oil a good starting material for many applications since the hydroxyl functionality makes it very suitable for isocyanate reactions such as those mentioned above [15]. Unfortunately, castor oil has been classified as a semi-drying oil. This means that those materials incorporating castor oil in their structure will never achieve complete curing through oxidative crosslinking, as they would if they incorporated oils with many double bonds in each fatty acid moiety [16]. For this reason, castor oil substitution also needs to be addressed and biomass-derived polyols could represent a good alternative. 

Herein, we report the preparation of wheat straw polyols through a liquefaction reaction, as well as its use for the preparation of biodegradable polyurethane foams by substituting castor oil. In addition, the influence of the structure of the isocyanate used during foam formulation is studied in order to elucidate the optimum composition for the prepared foams, addressing the maximum castor oil substitution. Therefore, biodegradability studies of the prepared foams have been developed for future applications in plant nurseries.

## 2. Materials and Methods 

### 2.1. Materials

The wheat straw used in this study was provided by a local farmer in Cordoba, Spain. The raw material was conditioned up to constant moisture, ground in a hammer mill, sieved to obtain a 4–6 cm fraction free of impurities, and characterized according to standard methods [17]: 39.7% cellulose; 30.6% hemicelluloses; 17.7% lignin; 7.7% ash, and 5.2% alcohol extractable. All determinations were performed by triplicate and the mean value was reported.

Reagents used during the liquefaction of wheat straw were glycerol (labkem, > 99% purity), and H_2_SO_4_ (PanReac, 95% purity, Barcelona, Spain). Employed reagents for the production of polyurethane foams were castor oil (Sigma Aldrich, St. Louis, MO, USA), dibutyltin dilaurate (Sigma Aldrich, 95% purity, St. Louis, MO, USA), silicone oil (Sigma Aldrich, St. Louis, MO, USA), methane di-p-phenyl diisocyanate (Sigma Aldrich, 98% purity, St. Louis, MO, USA), and toluene-2,4-diisocyanate (Sigma Aldrich, 80% purity, St. Louis, MO, USA). All other reagents used in this study were of analytical grade.

### 2.2. Liquefaction of wheat straw

In a typical liquefaction procedure, 20 g of dried and milled wheat straw was loaded into a 500 mL glass reactor equipped with a mechanical stirrer, temperature control, and condenser. The raw material was mixed with 100 mL glycerol and 3 g of H_2_SO_4_ (catalyst) and heated until 160 °C for 1 h under constant stirring (300 rpm). These reaction conditions have been optimized by the research group in previous experiments [18,19]. After the residence time, the glass reactor was immersed in cold water to quench the reaction. The liquefied fraction was filtered to remove the un-reacted solid residue. The residue was washed with acetone several times, dried at 105 °C for 12 h in a ventilated oven, and weighed to determine the reaction yield (*η*) as a measure of a liquefaction reaction extent:

$$\eta =\;\left[1-\;\frac{M}{M_{0}}\right]\;\times \;100$$

where *M*_0_ is the mass of initial dried wheat straw and *M* is the mass of the final residue obtained after the liquefaction process.

### 2.3. Preparation of Polyurethane Foams

Polyurethane (PU) foams were synthesized in 370 mL plastic cups using the one-shot method. Polyol from wheat straw liquefaction (LWS) and castor oil were mixed into different proportions to evaluate the grade of substitution (bio-polyol vs. synthetic oil). The foaming formulation also included dibutyltin dilaurate as a catalyst, distilled water as a blowing agent, and silicone oil as a surfactant. The amounts of these components were kept constant to evaluate only the substitution possibility of castor oil by LWS. Finally, two different isocyanates, MDI (methylene diphenyl diisocyanate) and TDI (toluene-2,4-diisocyanate), were tested in the foam formulation (Table 1). It should be mentioned that the maximum grade of substitution achieved was 50%, as it was not possible to obtain stable foams with higher substitution values.

During the preparation, LWS, castor oil (CO), blowing agent (0.4 g), surfactant (0.4 g), and catalyst (0.4 g) were mixed for 1 min at 1000 rpm using mechanical stirring. Isocyanate was then added and remained under stirring for 20 s at 1000 rpm. Finally, the contents of the plastic cups were allowed to foam and settle for a week prior to analysis. 

During the foam production some physical properties were measured (Table 1). Cream time is the time in seconds where the mixture begins to change from liquid to a creamy state before expansion. Free rise time is the time in seconds from the expansion starting point to the maximum height of the foam [20].

The NCO/OH molar ratio (R*_NCO/OH_*) was calculated following the method described by Kim et al. [21] as reported in a previous study [22]:

$$R_{NCO/OH}=\frac{\frac{Isocyanate\;\left(g\right)\;\times \;2}{MW_{Isocyanate}}}{\frac{H_{2}O\;\left(g\right)\;\times \;2}{MW_{H_{2}O}}+\;\frac{LWS\;\left(g\right)\;\times \;33.56}{MW_{LWS}}+\;\frac{CO\;\left(g\right)\;\times \;9.06}{MW_{CO}}}$$

### 2.4. Characterization of Products

#### 2.4.1. Liquefied Product

The pH was measured using 1 g of liquefied product dissolved in 50 mL of water. The mixture was vigorously stirred, and the pH was recorded in triplicate using a calibrated pH meter (Crison GLP 31, Barcelona, Spain). 

Viscosity measurements were carried out at 20 °C using a rotatory viscometer (Selecta ST-2020, La Rioja, Spain). 

The molecular weight distribution of LWS was determined by size-exclusion chromatography (SEC) in a PL-GPC 50 integrated gel permeation chromatography (GPC) system (Agilent Technologies, Madrid, Spain) at 50 °C, equipped with a refractive index (RI) detector, a KD-G 4A guard column (Shodex, Prague, Czech Republic), and a KD-806 M column (Shodex). 

The hydroxyl number (I_OH_) was determined following the American Society for Testing and Materials (ASTM) D4274–16 standard [23]. It consisted of dissolving a weighed amount of liquefied product in an acetylation reagent (acetic anhydride in pyridine) and the mixture was heated in a water bath at 98 °C for 2 h. The acetic acid formed was titrated with 0.5 N NaOH solution. The I_OH_ was corrected using the acid number due to the acid character of LWS. LWS was dissolved in a 50 mL mixture of dioxane and water (4:1 v/v), phenolphthalein (1% ethanol) was added, and the solution was titrated with 0.1 M KOH. Both the I_OH_ and acid number of the samples were obtained by the difference in titration between the blank and the sample solutions. All the determinations were repeated in triplicate and standard deviation is shown.

#### 2.4.2. LWS-Formulated PU Foams

The densities of the foam samples were determined according to ASTM D1622-14 [24]. The compressive strength was determined in general accordance with ASTM D1621-16 [25], on a universal testing machine (Instron Tecsis 4468) with a load cell of 50 kN at the cross-head speed of 2.5 mm/min. The load was parallel to the foam rise direction. The specimens were those used in the apparent density measurement. The compressive strength was the stress at the yield point (if before 10% deformation) or the stress at 10% deformation. The reported results are averages of at least three samples [26].

Scanning electronic microscopy (SEM) was carried out on the obtained foams to observe and evaluate the cell morphology of the foams. The imaging was performed with a JEOL JSM-6300 scanning microscope equipment operated at 20 kV and beam currents between 0.01 and 0.1 nA. The cell size of the prepared foams was measured using ImageJ. 

Thermal properties of PU foams were analyzed using a TA Instrument TGA Q50 thermogravimetric analyzer (Mettler-Toledo, Barcelona, Spain). The measurements of samples’ weight loss in relation to the temperature of thermal degradation were carried out between 50 and 800 °C at 10 °C/min under a N_2_ flow (20 mL/min).

The determination of the biodegradability of PU foams was conducted according to ASTM D5988-03 [27]. The soil medium used for the test was leaf soil from the nursery of the University of Bío bío, with a humidity of 62%. The foams were cut to an area of 1 cm^2^ and weighed (*W_i_*). Each foam was then buried 2 cm deep in a polypropylene plastic capsule, where the substrate was located. After 30 and 60 days, the foams were dug up, washed with distilled water, dried in an oven at 50 °C for 24 h and weighed (*W_f_*). The percentage of biodegradability (%*D*) was calculated as the difference between the initial and final weights of the samples according to the following equation:

$$\%D=\frac{W_{i}-W_{f}}{W_{i}}\times 100$$

## 3. Results and Discussion

### 3.1. Chemical Composition of Wheat Straw

The chemical composition of wheat straw with 39.7% cellulose and 30.6% hemicelluloses ensures a high availability of hydroxyl groups, being a carbon and hydrogen rich feedstock, apt for success in the liquefaction reaction process. Previous studies of the research group, using a great variety of agricultural residues with similar composition, gave us enough experience to carry out the wheat straw liquefaction.

The high ash content (7.7%) was lower than other similar straws such as rice straw (15.2%) or corn straw (12.4%) [18], but it could cause some yield problems during the liquefaction reaction.

### 3.2. Wheat Straw Liquefaction and Product Properties

The liquefaction reaction carried out for wheat straw enabled a high yield to be obtained, which was close to the maximum (Table 2). This suggested that, as expected, the large amount of hydroxyl groups (from cellulose and hemicelluloses) ensured the success of the reaction. During the liquefaction process, two reactions occur. When the first one (degradation) predominates, the greatest decomposition of the raw material takes place and, therefore, the amount of unconverted material is reduced, thus increasing the yield. However, when the second reaction (repolymerization) predominates, the solid residue content increases. In the case of LWS, it seems that the first reaction predominated, which is why such high yields were obtained. This result is due to the chosen reaction conditions optimized by the research group in previous research [18,19]. 

Considering the proposed application for LWS (PU foam preparation), the hydroxyl number, viscosity, and molecular weight are the most important parameters to consider. LWS exhibited a hydroxyl number of 604.1 mg KOH/g. Polyols from petrochemical origin, usually employed in industry, present a hydroxyl number of about 200 mg/g [28]. Polyols from natural oils studied in the literature present slightly higher hydroxyl number values. For example, Kurańska and Prociak [28] reported 276 mg KOH/g for rapeseed-oil-based polyol. Similarly, Zlatanić et al. [29] reported 207.0, 231.4, and 237.1 mg KOH/g for canola, soybean, and sunflower-oil-based polyols, respectively. When it comes to castor oil, it has a higher value (408 mg KOH/g) with similar liquefaction yields (92.54%) [15]. In these cases, a low hydroxyl number was related to high viscosity since viscosity values were 2.94, 3.35, and 4.08 Pa·s for canola, soybean, and sunflower-oil-based polyols [29]. Castor-oil-based polyol, with a high hydroxyl number, exhibited 16.4 Pa·s [15]. These oils were all successfully used for the preparation of polyurethane foams, with castor oil being the one that provided the best foams. These findings suggest that a high hydroxyl number, such as that obtained for LWS, is very desirable for the preparation of polyurethane foams. 

### 3.3. LWS-PU Foam Properties

The as-obtained LWS was used to prepare polyurethane foams as a substitute for castor oil, also studying the influence of the isocyanate used in the process. To this end, two different isocyanates were tested: TDI and MDI. These isocyanates, usually used in industry, have different structures that can influence the process of foam formation. Thus, TDI, with an aromatic ring, presents an asymmetry that disturbs the structure of the hard segment. This event is not found in MDI, which has two aromatic rings since it does not present the same isometric asymmetry [22]. 

With both isocyanates, it was possible to replace the castor oil up to a maximum of 50%. Foams formulated with TDI exhibited R_NCO/OH_ from 0.554 to 0.867. In the case of MDI, as expected due to its higher molecular weight, the range was slightly lower since the blank sample (BM) exhibited R_NCO/OH_ of 0.386 and the 50:50M foam R_NCO/OH_ of 0.603. If cream and free rise times are considered (Table 1), these both increased with a greater amount of polyol in the structure. This is because more polyol in the system requires more time to start the gelling and blowing reactions. Again, differences in cream and free rise times between TDI- and MDI-formulated foams were observed, with the latter being considerably shorter. In other words, lower R_NCO/OH_ meant lower cream and free rise times. This event was described by Zhang et al. [30] for polyurethane foams prepared with oilseed rape straw, rice straw, wheat straw, and corn stover, where the higher the R_NCO/OH_, the shorter the foaming times. 

**Figure 1:**
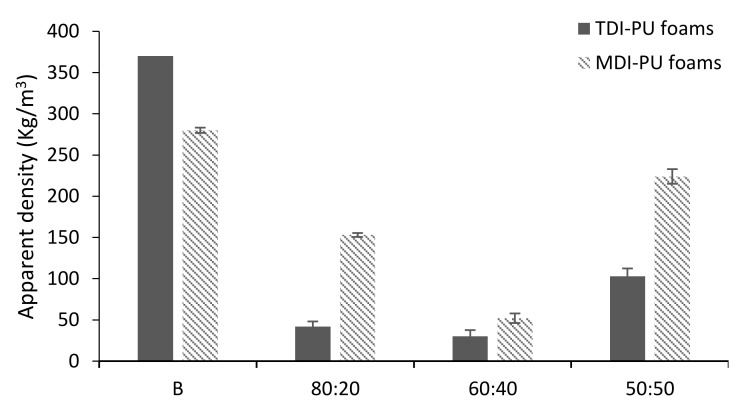
Apparent density of wheat straw liquefaction (LWS)-formulated polyurethane (PU) foams.

Apparent densities of the TDI- and MDI-LWS foams are displayed in Figure 1. In the case of the TDI foams, the blank sample (BT) presented a very high apparent density value (370.1 Kg/m^3^, Table S1). We have previously reported that this effect is due to a shorter cure rate than gas formation rate, which promotes a collapse of the foam before curing [22]. For this reason, we consider that the data obtained from this sample should not be used, since the sample obtained was as hard as a stone, instead of the consistency of a foam. The rest of the foams formulated with TDI presented apparent densities in the range 30–105 Kg/m^3^. When LWS was present in the foam at 20 and 40%, the densities were very similar (R_NCO/OH_ of 0.647 and 0.779, respectively). However, as the amount of polyol in the system increased to 50%, the density more than doubled (103.0 Kg/m^3^, Table S1). Foams 80:20T and 60:40T exhibited density values similar to those foams prepared with oilseed rape straw and rice straw [30]. However, these foams were prepared with a R_NCO/OH_ considerably higher (1.0) than those reported in the present study. Similarly, castor-oil-based PU foams presented density values between 36.0–36.8 Kg/m^3^ [15]. This means that to obtain the same density value, the presence of LWS replacing castor oil achieves the same result but with a significantly lower R_CNO/OH_.

On the other hand, in general, the foams prepared with MDI showed much higher apparent density values (in the range 50–280 Kg/m^3^) with R_NCO/OH_ slightly lower than the TDI foams. However, MDI foams did not exhibit the slightly upward trend in density with the amount of polyol in the system that was observed in TDI foams. In this case, the density decreased from the blank sample to 60:40 foam, then increased slightly to 50% LWS in the system. This same trend has been reported for PU foams formulated with epoxidized rapeseed oil, where by increasing the amount of polyol in the material, the described density fluctuation was observed [31].

**Figure 2:**
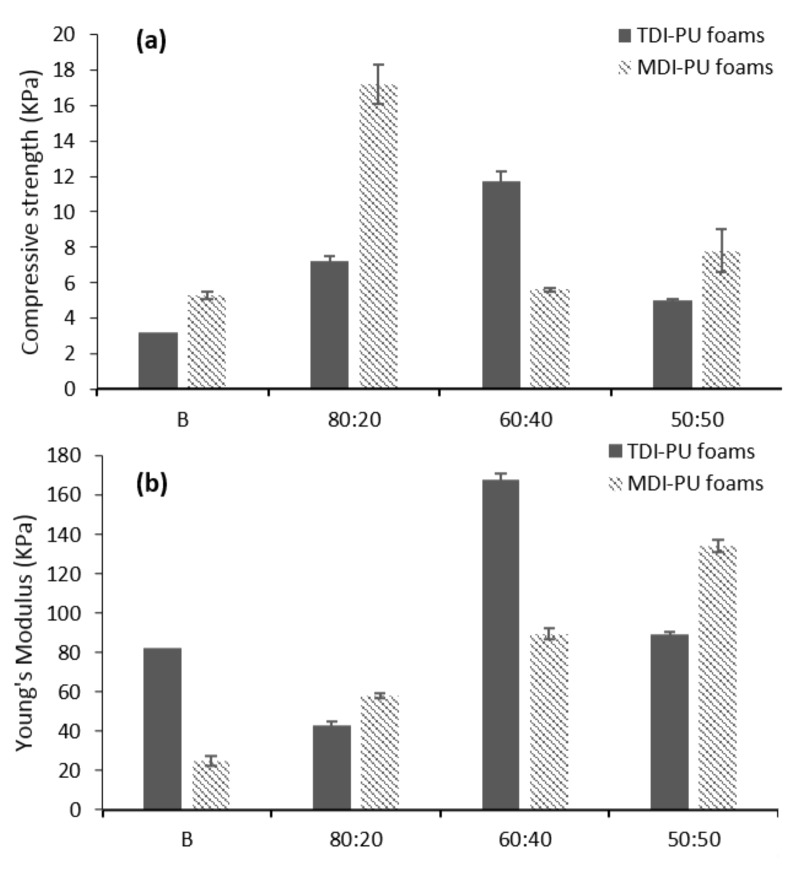
Mechanical properties of LWS-formulated PU foams: (**a**) Compressive strength and (**b**) Young’s Modulus.

Mechanical properties results are shown in Figure 2. In the case of TDI foams, they showed increasing compression strength values as the amount of LWS in the foam increased, up to 40% substitution (from 3.2 KPa in BT to 11.7 KPa in 60:40T). As the LWS in the system increased to 50%, the compression strength decreased to 5 KPa (Table S1). 

MDI foams again showed a fluctuating trend where the compression strength increased for the 80:20M sample (17.2 KPa) with respect to BM (5.3 KPa), then decreased considerably with 40% LWS and increased again slightly for 50:50M foam (7.8 KPa). The compressive strength values obtained confirmed that the prepared foams were of a flexible type, which is usually classified according to the mechanical performance. Flexible PU foams owe their properties to phase separation: hard segment and soft segment. The hard segment would be the zone that physically intercrosses, giving firmness to the polymer, while the soft segment is made up of stretchable chains that give the polymer elasticity [32]. 

Regarding the Young’s modulus, the 80:20T sample was the one exhibiting the best result with 167.5 KPa. From then on, the value started to decrease presenting 89.3 KPa for 50:50T sample. In contrast, the foams formulated with MDI presented higher Young’s moduli as the amount of LWS increased (from 24.9 KPa in BM to 134.2 KPa in 50:50M). If these data are compared with those of flexible foams prepared with castor oil, it can be observed that, both using TDI and MDI, the values obtained in the present work increase more than ten times from those described in the literature [33].

**Figure 3:**
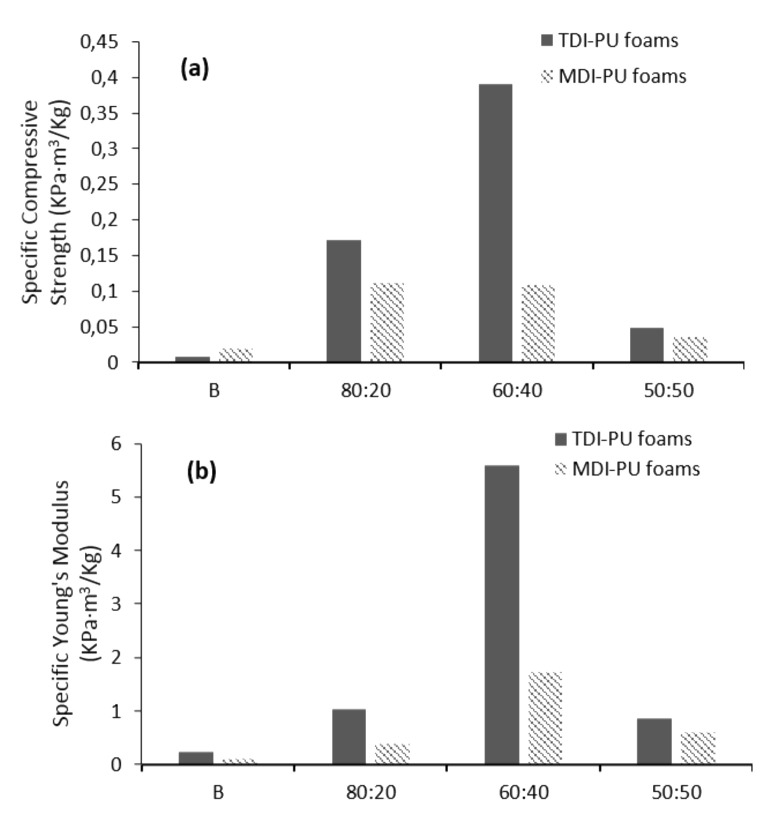
Specific (**a**) compressive strength and (**b**) Young’s Modulus of LWS-formulated PU foams.

As apparent density values vary between the foam samples, mechanical properties were calculated avoiding the effect of the density. In this way, specific mechanical properties were obtained and are displayed in Figure 3. As observed, 60:40T is still the TDI foam with the best result, both in compressive strength and Young’s modulus. Interestingly, this calculation helped to determine which MDI foam was the optimal. At equal compressive strength, the Young’s modulus was considerably higher for the 60:40M sample. 

Considering the results obtained in the evaluation of apparent density and mechanical properties, it could be stated that castor oil can be replaced by LWS up to 40%, maintaining its properties, regardless of the isocyanate used. 

**Figure 4:**
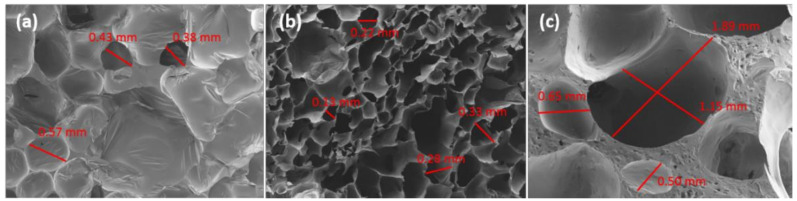
SEM images of (**a**) 80:20T, (**b**) 60:40T, and (**c**) 50:50T foams.

The morphology of the cells in the prepared foams was studied by SEM. As mentioned above, the high hardness of the blank sample prepared with TDI (BT) made this analysis impossible for that sample. The SEM images of the other LWS-TDI-formulated foams are shown in Figure 4. When LWS was present at 20% (80:20T), the cell morphology was found to be amorphous, as cell diameters from 0.38 to 0.84 mm were present. When the presence of LWS was increased to 40% (60:40T), the structure found was very uniform and ordered, with an average cell diameter of 0.56 ± 0.11 mm. When the maximum degree of castor oil substitution was reached (50:50T), the cell structure reappeared as disordered and with very variable sizes from 0.50 mm to 1.89 mm. This fact has been previously described by other authors, who claim that the difference in cell morphology when the amount of polyol increases is due to the increase in viscosity [30]. As the size of the cell increases, holes appear in the structure causing lower performance of these foams. In addition, the increase in R_NCO/OH_ (0.779 and 0.867 for 60:40T and 50:50T, respectively) slows the release of CO_2_ during the formation of the foams, forming a compact layer and causing the concentrated gas to break through the cell wall and form holes [22]. In addition, if the morphology obtained is contrasted with the mechanical properties previously evaluated, it can be observed that there is a perfect correlation. The foam formulated with TDI that presented the best mechanical performance (60:40T), was the same one that exhibited an orderly and compact cell morphology.

**Figure 5:**
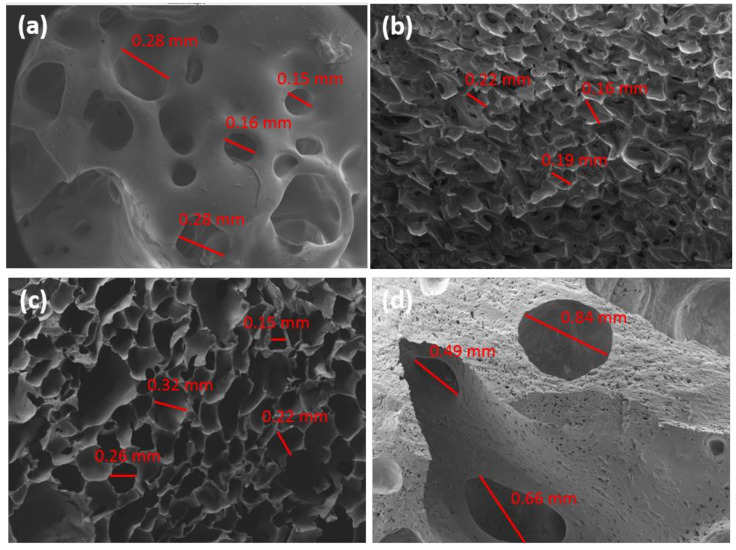
SEM images of (**a**) BM, (**b**) 80:20M, (**c**) 60:40M, and (**d**) 50:50M foams.

SEM micrographs of LWS-MDI-formulated foams are shown in Figure 5. As with the foams formulated with TDI, MDI-formulated foams showed an ordered cell structure for 80:20M and 60:40M formulations (cell diameter of 0.28 ± 0.04 mm in both cases). These, in turn, had the best mechanical properties. When dealing with foams prepared with vegetable oils, the cells are usually arranged and reduced in size as the amount of bio-polyol in the structure increases, as reported by Tu et al. [34] for PU foams prepared with soybean-oil-based polyol. 

The foams formulated with castor oil that have been evaluated in the literature show a very similar morphology to those presented in this work, with cell diameters between 0.12 and 0.5 mm. Therefore, the data obtained suggest that LWS could be a very suitable candidate to replace castor oil in the formulation of PU foams [35].

**Figure 6:**
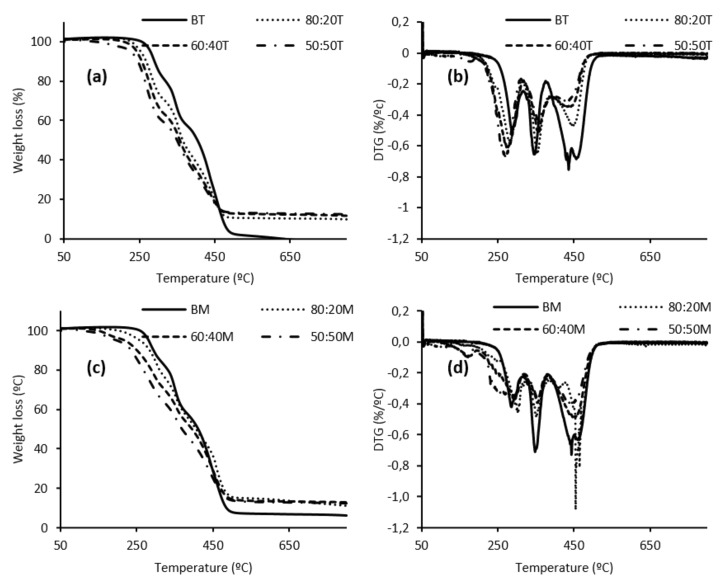
(**a**)TGA and (**b**) DTG curves of foams prepared with TDI. (**c**) TGA and (**d**) DTG curves of foams formulated with MDI.

Finally, prior to the application proposed for the LWS-formulated foams, the thermal properties were evaluated by TGA. Thus, the TGA and derivative thermogravimetric (DTG) curves of the TDI foams are shown in Figure 6.

As explained in a previous work on PU foams with a laurel tree pruning waste-based polyol, the degradation mechanism of PU foams is complicated. Many degradation products are formed resulting in differential weight loss curves with many peaks [22].

Thus, TDI foams showed three main decomposition peaks (T_1_, T_2_, and T_3_ in Table S2). The first peak of degradation, associated with the loss of volatile compounds, is between 264–289 °C. The T_2_ peak, attributed to the breakdown of the urethane links of the polymer, is the range 354–356 °C. The last peak of degradation, T_3_, due to the decomposition of LWS, is in the range 436–447 °C. The very weak variation in temperature ranges of the decomposition peaks was not significant between the different TDI-LWS foams. However, it should be noted that the first stage of decomposition is quite significant between LWS foams and the reference foam (BT). Thus, the blank sample remains without weight loss until 257 °C, while foams incorporating 20% LWS begin to lose weight at 228 °C, an event that intensifies as the amount of LWS in the system increases (50:50T sample begins to lose weight at 208 °C). Therefore, it could be said that, despite having a slightly lower resistance to thermal degradation than the blank, TDI foams have good thermal characters since they remain stable up to 200 °C.

TGA and DTG curves of LWS-MDI foams are also presented in Figure 6. In this case, again three main degradation peaks were found (Table S2). The ranges of the three peaks were slightly higher than for the foams formulated with TDI (297–302 °C, 348–353 °C, and 447–457 °C for T_1_, T_2_, and T_3_, respectively). This was an indication that the foams formulated with MDI had a slightly higher resistance to thermal degradation than TDI. This may be because R_NCO/OH_ of these foams was lower than that of the TDI foams. Although this decreased R_NCO/OH_ indicates that fewer urethane bonds will be formed in MDI foams, the structural differences between these two diisocyanates should be considered. It seems that the presence of two aromatic rings in MDI, rather than one as in TDI, in addition to the lack of isometric asymmetry of this compound, promotes the slower thermal degradation in MDI foams. Castor-oil-formulated foams in the literature also show the three typical degradation peaks. However, the temperatures of these events are lower (223.7, 324.0, and 487.5 °C, for T_1_, T_2_, and T_3_, respectively). This indicates that LWS is able to successfully replace castor oil in PU foams by not only maintaining but also improving the properties of the foams [36].

### 3.4. LWS-PU Foam Application: Biodegradability in Soil Media

**Figure 7:**
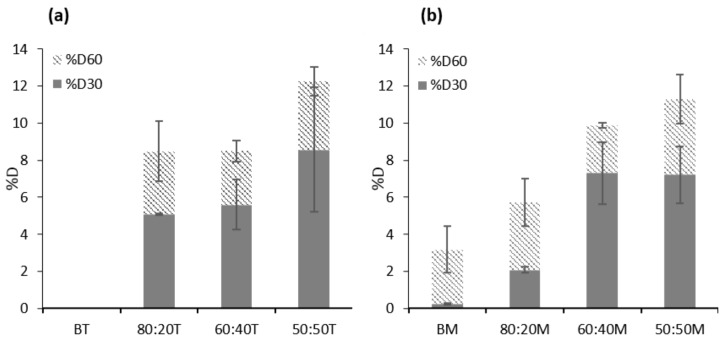
Percentage of biodegradability of (**a**) TDI LWS-PU foams and (**b**) MDI LWS-PU foams after 30 days (%*D*_30_) and 60 days (%*D*_60_).

PU foams of petrochemical origin are non-biodegradable materials. Therefore, the development of PU foams that incorporate bio-polyols such as LWS should not only match or improve the mechanical properties or morphology of the former but should also be biodegradable. This is how the possible replacement of PU by active polyols derived from natural resources could be stated. In this sense, the foams prepared in this work were subjected to biodegradability tests over 30 (%*D*_30_) and 60 days (%*D*_60_), obtaining the results shown in Figure 7. 

In the case of TDI-formulated foams, it was observed that the biodegradability, both at 30 and 60 days, was very similar in the case of 80:20T (5.09 and 8.48% of %*D*_30_ and %*D*_60_, respectively) and 60:40T (5.60 and 8.49% of %*D*_30_ and %*D*_60_, respectively) foams (Table S2). The most remarkable increase in biodegradability was the 50:50T foam that presented 8.57% at 30 days and 12.25% at 60 days. MDI-formulated foams exhibited a moderate increasing trend with increasing LWS substitution for %*D*_30_. Thus, 60:40M foam exhibited 7.31% biodegradability, an increased property compared to the blank sample (BM 0.23%) and the 80:20M foam (2.08%). From 40% LWS in the material, the biodegradability at 30 days was maintained, despite increasing the amount of polyol in the system (7.21% for 50:50M). When biodegradability at 60 days was studied for these same foams, it was found that this property was directly related to the amount of LWS. Thus, the most substituted foam (50:50M) presented 11.28%, a much higher result than the blank (3.18%). 

If the results obtained in this study are compared with foams formulated with vegetable oils, it can be stated that LWS significantly improved the biodegradability of the polymers. The foams with soybean oil showed less than 7.5% biodegradability after 100 days of testing. The same applies to foams formulated with castor oil that exhibit less than 5% biodegradability after 100 days [37]. Foams formulated entirely with castor oil usually have such low biodegradability due to their hydrophobic nature (long chains of aliphatic acidic hydrocarbons). Therefore, water molecules cannot penetrate the matrix and reach the hydrolytically labile ester groups of the castor oil. In our case, as the amount of LWS replacing castor oil increases, the biodegradability of the material increases. A possible explanation for this is the increase of more polyethylene glycol (PEG) segments from the polyol. These segments provide more water molecules in the environment of the ester groups, accelerating the degradation reaction and thus increasing the biodegradability [38]. 

## 4. Conclusions

Wheat straw residues were used to produce a natural bio-polyol by means of a highly optimized liquefaction procedure, which reported an excellent yield (96.5 %). This polyol exhibited excellent properties for the preparation of flexible polyurethane foams (I_OH_ 604.1 mg KOH/g and viscosity 0.6 Pa·s) where a vegetable oil such as castor oil was substituted by the liquefied product up to 50% *wt*. Additionally, the as-mentioned polyurethane foams were prepared using two different isocyanates (MDI and TDI) studying their influence in the final polymer structure.

Foams prepared with both TDI and MDI achieved their optical physical and mechanical properties with 40% bio-polyol substitution (specific compressive strengths of 0.390 and 0.108 KPa·m^3^/Kg, and Young’s moduli of 5.583 and 1.719 KPa·m^3^/Kg for TDI and MDI foams, respectively). These same foams presented an orderly and compact cell structure as well as improved thermal characters.

Finally, biodegradability assays showed that by increasing the amount of wheat straw polyol in the polymer foams, the biodegradability was also improved after 30 and 60 days. Furthermore, this property was increased up to 40% of polyol substitution in the system, being maintained with higher quantities. In this way, results obtained through this study encourage the use of wheat straw polyol as an efficient substituent for castor oil in polyurethane foams, which can be used in plant nurseries. 

## Figures and Tables

**Table 1:** Foam formulations and nomenclature.

Sample Name	Formulation (CO: LWS)	CO (g)	LWS (g)	TDI (g)	MDI (g)	R*_NCO/OH_*	Cream Time (s)	Free Rise Time (s)
BT	Blank	20	0	11.5	0	0.554	42	72
80:20T	80:20	16	4	11.5	0	0.647	50	98
60:40T	60:40	12	8	11.5	0	0.779	111	173
50:50T	50:50	10	10	11.5	0	0.867	102	148
BM	Blank	20	0	0	11.5	0.386	5.36	14.87
80:20M	80:20	16	4	0	11.5	0.451	7.43	25.96
60:40M	60:40	12	8	0	11.5	0.542	24.94	59.81
50:50M	50:50	10	10	0	11.5	0.603	51	72

**Table 2:** Wheat straw polyol properties.

Sample	Yield (%)	pH (25 °C)	I_OH_ (mg KOH/g)	Acid number (mg KOH/g)	Viscosity (Pa·s)	Mw (g/mol)	Mn (g/mol)
LWS	96.5	1.63 ± 0.02	604.1 ± 9.1	59.2 ± 0.86	0.6 ± 0.05	30,463	28,170

## Supplementary Materials

The following are available online at https://www.mdpi.com/2073-4360/12/11/2646/s1, Table S1: Apparent density and mechanical properties of LWS-PU-foams, Table S2: Thermal characters and biodegradability of LWS-formulated foams.
