# Supplementary material for: Bio-Degradable Polyurethane Foams Produced by Liquefied Polyol from Wheat Straw Biomass
Source: Polymers (Basel). 2020 Nov 10;12(11):2646. doi: 10.3390/polym12112646 (PMC7709019; doi:10.3390/polym12112646)
Supplement: Supplementary file 1 [file polymers-12-02646-s001.zip › polymers-989425-supplementary.docx]

**Bio-degradable polyurethane foams produced by liquefied polyol from wheat straw biomass.**

**Luis Serrano ^1,^*, Esther Rincón ^1,2^, Araceli García ^2^, Jesús Rodríguez ^3^, and Rodrigo Briones ^3^**

^1^ Inorganic Chemistry and Chemical Engineering Department, University of Cordoba, 14014, Cordoba, Spain; [b32rirue@uco.es](mailto:b32riruel@uco.es) (E.R.)

^2^ Organic Chemistry Department, University of Cordoba, 14014, Cordoba, Spain; [qo2ganua@uco.es](mailto:qo2ganua@uco.es) (A.G.)

^3^ Centro de Investigación de Polímeros Avanzados (CIPA), Av. Collao 1202, Concepción, Chile; [j.rodriguez@cipachile.cl](mailto:j.rodriguez@cipachile.cl) (J.R.); [r.briones@cipachile.cl](mailto:r.briones@cipachile.cl) (R.B.)

***** Correspondence: [luis.serrano@uco.es](mailto:luis.serrano@uco.es) (L.S.)

**SUPPLEMENTARY DATA**

**Table S1**. Apparent density and mechanical properties values of LWS-PU-foams.

| Sample | Apparent density (Kg/m^3^) | Compressive strength (CS, KPa) | Young’s Modulus (YM, KPa) | Specific CS (KPa·m^3^/Kg) | Specific YM (KPa·m^3^/Kg) |
| --- | --- | --- | --- | --- | --- |
| BT | 370.1 ± 0.0 | 3.2 ± 0.0 | 82.3 ± 0.0 | 0.009 | 0.222 |
| 80:20T | 42.0 ± 6.1 | 7.2 ± 0.0 | 43.1 ± 1.8 | 0.171 | 1.026 |
| 60:40T | 30.0 ± 7.6 | 11.7 ± 0.0 | 167.5 ± 3.5 | 0.390 | 5.583 |
| 50:50T | 103.0 ± 9.4 | 5.0 ± 0.0 | 89.3 ± 1.4 | 0.049 | 0.867 |
| BM | 280.0 ± 3.2 | 5.3 ± 0.0 | 24.9 ± 2.2 | 0.019 | 0.089 |
| 80:20M | 153.0 ± 2.4 | 17.2 ± 0.0 | 58.1 ± 1.4 | 0.112 | 0.380 |
| 60:40M | 52.0 ± 5.8 | 5.6 ± 0.0 | 89.4 ± 2.7 | 0.108 | 1.719 |
| 50:50M | 224.0 ± 8.9 | 7.8 ± 0.0 | 134.2 ± 3.2 | 0.035 | 0.559 |

**Table S2**. Thermal characters and biodegradability of LWS-formulated foams.

| Sample | T_1_ (ºC) | T_2_ (ºC) | T_3_ (ºC) | Final residue (%) | *%D*_30_ | *%D*_60_ |
| --- | --- | --- | --- | --- | --- | --- |
| BT | 284.00 | 354.33 | 436.33 | 0 | - | - |
| 80:20T | 280.33 | 354.33 | 447.83 | 9.86 | 5.09 ± 0.06 | 8.48 ± 1.68 |
| 60:40T | 278.50 | 354.33 | 441.33 | 11.67 | 5.60 ± 1.34 | 8.49 ± 1.90 |
| 50:50T | 269.87 | 356.33 | 438.67 | 12.39 | 8.57 ± 3.37 | 12.25 ± 2.58 |
| BM | 300.00 | 348.17 | 457.57 | 6.20 | 0.23 ± 0.05 | 3.18 ± 1.32 |
| 80:20M | 302.17 | 351.50 | 454.50 | 11.15 | 2.08 ± 0.15 | 5.72 ± 1.43 |
| 60:40M | 302.17 | 353.33 | 453.17 | 12.91 | 7.31 ± 1.66 | 9.88 ± 1.81 |
| 50:50M | 297.00 | 353.33 | 447.67 | 12.38 | 7.21 ± 1.55 | 11.28 ± 2.87 |
